# CT Patterns of Interstitial Lung Disease in Patients with Plaque Psoriasis: A Retrospective Case Series Study

**DOI:** 10.3390/medicina59091650

**Published:** 2023-09-12

**Authors:** Giulio Rizzetto, Corrado Tagliati, Marco Fogante, Matteo Marcucci, Giulio Argalia, Giuseppe Lanni, Alberto Rebonato, Gian Marco Giuseppetti, Roberto Esposito, Elisa Molinelli, Edoardo De Simoni, Annamaria Offidani, Oriana Simonetti

**Affiliations:** 1 Department of Clinical and Molecular Sciences, Dermatology Clinic, Polytechnic Marche University, 60121 Ancona, Italy; g.rizzetto@pm.univpm.it (G.R.); edodesimoni@hotmail.it (E.D.S.);; 2 Radiologia AST Pesaro Urbino, 611121 Pesaro, Italy; corradotagliati@gmail.com (C.T.); alberto.rebonato@ospedalimarchenord.it (A.R.); roberto.esposito@sanita.marche.it (R.E.); 3 Department of Radiological Sciences, Azienda Ospedaliero Universitaria Ospedali Riuniti, Università Politecnica delle Marche, 60121 Ancona, Italygiulio.argalia@gmail.com (G.A.); giuseppettigianmarco@gmail.com (G.M.G.); 4 U.O.S.D. Radiologia Ospedale “San Liberatore” Atri-Dipartimento dei Servizi-ASL Teramo, 64032 Teramo, Italy

**Keywords:** plaque psoriasis, interstitial lung disease, radiology, computed tomography

## Abstract

*Background and Objectives:* Recently published articles reported an association between psoriasis and interstitial lung diseases (ILDs). The aim of this study is to evaluate the differences in ILD computed tomography (CT) patterns between smoker and never smoker plaque psoriasis (PP) patients under topical treatment without psoriatic arthritis (PA), inflammatory bowel disease (IBD) or connective tissue diseases (CTDs). *Matherials and Methods*: Two radiologists evaluated chest CT examinations of 65 patients (33 smokers, 32 never smokers) with PP. *Results:* Usual interstitial pneumonia (UIP) pattern was diagnosed in 36 patients, nonspecific interstitial pneumonia pattern in 19, hypersensitivity pneumonitis in 7 and pleuropulmonary fibroelastosis (PPFE) in 3 patients. UIP pattern showed a statistically significant higher frequency in smoker patients (*p* = 0.0351). Respiratory symptoms were reported in 80% of patients. *Conclusions:* ILDs seems to represent a new comorbidity associated with psoriasis. Moreover, a statistically significant association between smokers and UIP pattern in PP patients is found. Respiratory symptoms should be evaluated in PP patients, in collaboration with a radiologist and a pneumologist. However, further studies are required to better understand the epidemiology of ILDs in PP patients.

## 1. Background and Objectives

Psoriasis is a chronic immune-mediated inflammatory dermatoses of the skin, and usually consist of recurrent indolent red, scaly, sharply demarcated, indurated plaques or patches, which present particularly over the extensor surfaces of knee and elbow, trunk, and scalp. Plaque psoriasis (PP) is the most common type of psoriasis, accounting for approximately 85% of all cases, and it affects about 2.5% of the population in Western countries [1,2,3,4,5].

In recent years, more and more cases of interstitial lung diseases (ILDs) in psoriatic patients have been reported in the literature [6,7]. ILDs refers to a group of several different lung disorders characterized by pulmonary parenchyma inflammation and/or fibrosis that can be idiopathic or associated with known causes, such as rheumatologic diseases [6]. Although many aspects are still unclear about the relationship between psoriasis and ILDs, according to the current data, most cases are associated with moderate to severe psoriasis treated with immunosuppressants, such as methotrexate and azathioprine, or biologic drugs, particularly anti-TNFα antibodies, such as infliximab and etanercept [6,7]. We also know that ILDs may occur more frequently in patients affected by psoriasis and other comorbidities, such as psoriatic arthritis (PA), connective tissue diseases (CTDs) and inflammatory bowel diseases [7,8].

More specifically, it is estimated that approximately 10% of patients with rheumatoid arthritis may develop clinically relevant ILDs, but sub clinic forms of ILDs can be observed on high-resolution chest computed tomography (CT) examination in almost 50% of patients with rheumatoid arthritis [9]. The concomitant presence of psoriasis and rheumatoid arthritis could, therefore, confound the actual role of psoriasis in the development of ILDs. On the other hand, another CTD, ankylosing spondylitis, is associated with the presence of ILDs in approximately 2% of patients. Ankylosing spondylitis shares more similarities to psoriatic arthritis and psoriasis than rheumatoid arthritis, since ankylosing spondylitis shows serological negativity for rheumatoid factor, and an inflammatory cytokine pattern based on increased production of tumor necrosis factor alpha (TNF-α) and interleukin (IL)-17A [9]. Based on these considerations, it is, therefore, not implausible that psoriasis may be associated with ILDs. From a histological point of view, the pathology of ILDs from patients with psoriasis or psoriatic arthritis have, in fact, been evaluated, revealing morphological similarities with other CTDs and interstitial pneumonia with autoimmune features [9].

Furthermore, we have to recognize that the possible association between ILDs and systemic treatment for psoriasis is consistent, considering that a previous study reported an ILDs incidence in about 2% of patients with psoriasis who needed systemic treatments, with a mean psoriasis area severity index (PASI) of 12 [8]. Among these patients, the most used systemic biological treatments were adalimumab, infliximab and ustekinumab [8]. Interestingly, some patients treated with anti IL-12/IL-23, after the diagnosis of psoriasis and ILD, showed improvement in both psoriasis and associated pulmonary disease activity. The severity of psoriasis also seems to correlate with the risk of developing ILDs and with the severity of pulmonary inflammation. In addition, certain forms of psoriasis, such as generalized pustular psoriasis, have been reported to be more frequently associated with the development of ILDs in comparison to psoriasis vulgaris, or plaque psoriasis [8].

The association between ILDs and psoriasis has also been reported in another study, evaluating the CT patterns of 21 ILD cases with antecedent or concomitant diagnosis of psoriasis or psoriatic arthritis [10]. However, the severity of psoriasis was not assessed in this study, which also included patients with previous systemic treatments (immunosuppressive and chemotherapeutic treatments), as well as patients with positive serological markers for autoimmune diseases. Finally, a recently published article reported surgical pathology findings of psoriatic patients with ILDs [9]. In this study, patients suffering from psoriatic arthritis and patients on systemic therapy were also included. In addition, patients with inverse psoriasis, with pustular palmoplantar psoriasis, and with scalp psoriasis were also part of the sample. On the other hand, patients with a history of smoking and pathological findings of smoke derived ILDs were excluded. Even in this study, there were no clear reports about the severity of psoriasis in patients who developed ILDs. However, many patients who required systemic therapy probably had moderate to severe psoriasis.

Since there is a clear association between moderate to severe psoriasis or psoriatic arthritis, mainly under systemic treatment, and ILDs, we decided to assess whether mild to moderate plaque psoriasis, or vulgar psoriasis, is also associated with ILDs. To the best of our knowledge, no previous studies evaluated exclusively psoriatic patients with only plaque psoriasis without psoriatic arthritis, connective tissue disorders or inflammatory bowel disease, and not under systemic therapy. Moreover, no previous studies evaluated the relationship between smoking status and CT ILD patterns in these patients, describing possible differences in CT patterns.

Accordingly, the aim of this study is to evaluate the effect of smoking status on CT patterns of ILDs in patients with plaque psoriasis under exclusively topical treatment and/or phototherapy without arthritis, IBD or CTDs.

## 2. Materials and Methods

### 2.1. Patient Selection

**Figure 1:**
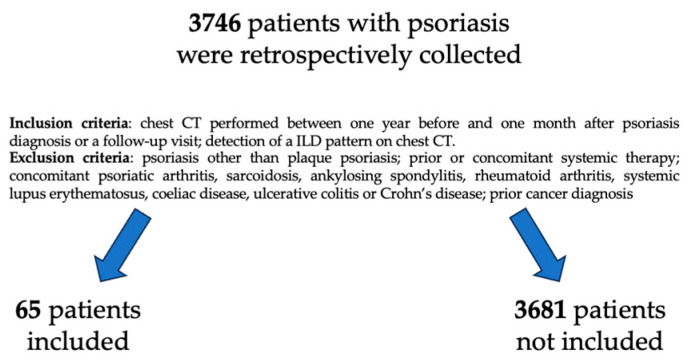
Flowchart of patients selected for the study.

From 1 January 2000 to 31 December 2019, 3746 patients with psoriasis were retrospectively collected in the Institution dermatologic database. Inclusion criteria were: chest CT performed at our hospital between one year before and one month after dermatologic visit for psoriasis; detection of a ILD pattern on chest CT. Exclusion criteria were: psoriasis other than plaque psoriasis (also known as psoriasis vulgaris); prior or concomitant systemic therapy; concomitant psoriatic arthritis, sarcoidosis, ankylosing spondylitis, rheumatoid arthritis, systemic lupus erythematosus, coeliac disease, ulcerative colitis or Crohn’s disease; prior cancer diagnosis. Patients with uncertain serology for connective tissue diseases were excluded as well. According to these criteria, 65 patients were included in this study (Figure 1). The study was approved by the institutional review board and ethics committee of our institution. Informed consent was obtained from all individual participants included in the study.

### 2.2. Plaque Psoriasis Diagnosis and Patient Clinical Assessment

As the clinical characteristics are usually sufficient to enable the diagnosis, plaque psoriasis diagnosis was made by experienced dermatologists on clinical grounds considering the presence of erythematous, scaling, and well demarcated plaques or patches, localized mostly on the extensor surfaces, trunk and head. Psoriasis area and severity index (PASI), body surface area (BSA) and static Physician Global Assessment (sPGA) on a 5-point rating were evaluated. Family history of psoriasis (first-degree relatives) was reported. Smoking status was assessed, and the patients were subdivided in two groups: never smokers, and current or former smokers, reported in the following sections as “smokers” [11]. Respiratory symptoms, such as chronic exertional dyspnea, shortness of breath or chronic cough, were assessed. Inclusion criteria considered only patients of 18 years of age or older, and with mild to moderate psoriasis, defined as sPGA < 3, PASI < 12, and body surface involvement < 10%. The presence of forms of psoriasis other than plaque psoriasis, such as pustular psoriasis, guttate psoriasis, erythrodermic psoriasis, inverse psoriasis, palmoplantar psoriasis was a clinical element of exclusion from the study.

### 2.3. Computed Tomography Examination

All CT examinations were performed using a 64 slices CT scanner (Lightspeed VCT 64, GE, Waukesha, WI, USA) with patients in supine position and head toward the gantry. After acquisition of an anterior–posterior topogram from neck to upper abdomen, CT examinations were performed in the caudocranial direction from costophrenic angles to lung apices in suspended deep inspiration. The scan parameters were as follows: 64 × 0.625 mm beam collimation, 120 kVp, 1.25 mm slice thickness and 1.25 mm slice thickness reconstructions, rotation time 0.5 s; matrix 512 × 512.

The acquisition parameters of the six evenly spaced axial expiratory Images obtained from 1 cm above the top of the aortic arch to 2 cm above the higher hemidiaphragm were: 120 kVp tube voltage, 40 mA tube current, 0.625 mm collimation, 0.5 s rotation time, matrix 512 × 512, slice thickness reconstruction of 1.25.

### 2.4. CT Images Evaluation

Two radiologists with at least 10 years of experience in chest computed tomography evaluated CT examinations in consensus.

Usual interstitial pneumonia (UIP) pattern included cases meeting criteria for ”UIP” and “probable UIP” by international consensus criteria [12,13,14]. Bilateral patchy bilateral and rather symmetrical ground-glass opacification with basal predominance and relative immediate subpleural sparing were used as criteria in order to diagnose a nonspecific interstitial pneumonia (NSIP) pattern [15,16,17]. A hypersensitivity pneumonitis (HP) pattern included cases meeting criteria for “typical HP” by ATS criteria [18]. Pleuropulmonary fibroelastosis (PPFE) pattern included cases meeting criteria for “definite” or “possible” PPFE by Lee et al.’s radiological criteria modified from Reddy et al.’s criteria and Enomoto et al.’ criteria [19,20,21].

### 2.5. Lung Biopsy

In total, 10 patients underwent lung biopsy in our institution performed by pneumologists, radiologist or thoracic surgeons with at least 10 years of experience in these procedures: 1 transbronchial forceps lung biopsy; 1 CT-guided transthoracic core lung biopsy; 8 transbronchial lung cryobiopsy, 7 of which performed subsequently video assisted thoracoscopic surgery.

### 2.6. Statistical Method

Qualitative variables frequency differences between plaque psoriasis smokers and never smokers were evaluated using a Chi-square test. Quantitative variables were evaluated using a *t*-test. The statistical significance level was set at *p* < 0.05. All statistical analyses were performed using MedCalc Software v. 15.8 (Ostend, Belgium, BEL).

## 3. Results

### 3.1. Clinical Findings

Patients’ characteristics are reported in Table 1. After applying the inclusion and exclusion criteria, 65 patients with a mean age of 63.9 ± 13.2 years, with a range of 29–88 years, were included in the study. Among these, 36 patients were male and 29 were female.

After dividing the included patients into two groups, smokers and never smokers, respectively, we observed no statistically significant differences in terms of age, *p* = 0.9032, sex, *p* = 0.9113, psoriasis area and severity index, *p* = 0.9236, body surface area of psoriasis, *p* = 0.4584, and static physician global assessment *p* = 0.6819. In addition, no statistically significant differences were also observed with regard to family history of psoriasis, *p* = 0.9714.

With regard to respiratory symptoms, we decided to classify them as a single characteristic because patients often presented with symptoms that fluctuated over time between chronic exertional dyspnea, shortness of breath, or chronic cough. Approximately 80% of the included patients experienced respiratory symptoms. However, we have additionally reported the percentage of individual respiratory symptoms that were recorded at the closest visit to the execution of pulmonary CT scan and the diagnosis of ILDs. Again, no statistically significant differences were recorded between smokers and never smokers in terms of shortness of breath or chronic exertional dyspnea (*p* = 0.5113) and chronic cough (*p* = 0.9317). The time of onset of respiratory symptoms was not considered because it was not clearly discernible from the retrospective analysis for all patients. We did not observe statistically significant differences in the frequency of respiratory symptoms between smokers and never smokers, *p* = 0.4951.

Finally, we evaluated pulmonary function tests (PFT), which showed no statistically significant differences between the smokers and never smokers groups in terms of forced vital capacity (FVC), *p* = 0.1744, forced expiratory volume in 1 s (FEV1.0), *p* = 0.6180, FEV1.0/FVC ratio, *p* = 0.1473, and lung diffusing capacity for carbon monoxide (DLCO), *p* = 0.1341.

### 3.2. Computed Tomography Findings

In total, 36 (55.4%) patients showed UIP pattern, 19 (29.2%) NSIP pattern, 7 (10.8%) HP pattern and 3 (4.6%) PPFE pattern. ILD CT patterns in plaque psoriasis patients current or former smokers and never smokers are reported in Table 2. Chi-square test showed UIP pattern statistically significant higher frequency in smoker patients (*p* = 0.0351).

Furthermore, we reported the typical CT findings of the ILDs detected in our patients. UIP radiological features were subpleural and basal predominant honeycombing or reticulation with peripheral traction bronchiolectasis. NSIP was mainly characterized by bilateral patchy areas of ground-glass attenuation with basilar distribution and relative immediate subpleural sparing. HP typical radiological findings were diffuse with possible basal sparing ill-defined centrilobular nodules and/or air trapping associated with ground glass opacities and/or mosaic attenuation with or without fibrosis. PPFE CT images showed upper lobes predominant pleural and subpleural thickening with a reticular pattern, with possible associated upper lobes volume reduction and traction bronchiectasis.

### 3.3. Lung Biopsy Findings

**Figure 2:**
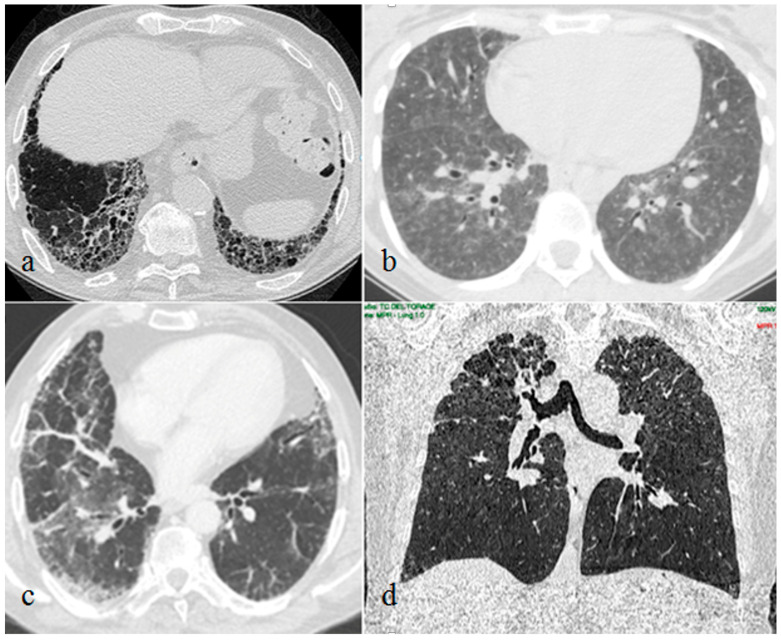
Interstitial lung disease patterns in patients with plaque psoriasis: usual interstitial pneumonia (**a**), hypersensitivity pneumonitis (**b**), nonspecific interstitial pneumonia (**c**), pleuropulmonary fibroelastosis (**d**).

The 10 lung biopsies performed confirmed the diagnosis suspected by the corresponding chest CT patterns. Seven patients showed NSIP, one patient showed PPFE, one patient showed HP and one patient showed early UIP pattern (Figure 2).

## 4. Discussion

Psoriasis is a chronic, inflammatory and immune-mediated skin disease, often associated with systemic manifestations. The most common is psoriatic arthritis, reported in about one third of cases, followed by inflammatory bowel disease, uveitis, lymphoma, metabolic syndrome, non-alcoholic fatty liver disease, hyperuricemia, gout, chronic obstructive pulmonary disease and psychiatric morbidity [22,23,24,25,26]. Therefore, psoriasis is an inflammatory disease which is not only limited to the skin, but also shows a potential systemic involvement, associated with numerous inflammatory conditions. Indeed, the skin production of pro-inflammatory cytokines can also affect the other organs, suggesting that a possible inflammatory involvement of the lung parenchyma is possible [8].

However, little is known about the relationship between psoriasis and ILDs. ILDs are a heterogeneous group of lung disorders, consisting of inflammation and/or fibrosis of the pulmonary parenchyma interstitium and peribronchovascular connective tissue [10]. CD4+ Th17 cells and their cytokine pathway may be the key to understand a possible correlation between ILDs and psoriasis. Therefore, Th17 cells have been implicated in the development of various fibrosing diseases of skin and lungs, including ILDs, by enhancing fibroblast proliferation and cytokine production, such as interleukin (IL)-17A, IL-17F, IL-21, IL-22, IL-6 and tumor necrosis factor-α (TNF-α) [27,28].

The IL-17 family cytokines are particularly important regulators of the mucosal immune response in respiratory tract, and an imbalance may lead to progressive fibrosis [29,30]. Furthermore, IL-17 receptor A (IL17RA) is also reported to play a direct role in lung fibrosis [31,32,33]. On the other hand, TNF-α, IL-23, IL-17 and IL-22 were reported to be fundamental in the pathogenesis of psoriasis [34,35]. More specifically, IL-22 showed an important role in the barrier surface integrity of skin, lungs and gut [36], while an increase in IL-22 was involved in airway remodeling in smokers [37]. Therefore, a possible shared underlying immunologic pathway between ILDs and psoriasis exists, although there are no specific studies yet [8,10,26,32].

In psoriatic patients, the incidence of ILDs seems related not only to risk factors, such as smoking, infections, obesity, air pollution, CTDs and certain systemic drugs, but also to the immunocytokine pattern of chronic psoriasis inflammation, particularly when associated with psoriatic arthritis [6]. In recent years, some studies and case reports evaluated the association between psoriasis and ILDs [38,39,40]. A case report showed that ILD activity fluctuated in parallel with psoriasis severity, supporting a possible causal link between psoriasis and ILDs [41]. Another case report showed the improvement of the interstitial lung pattern during psoriasis treatment with secukinumab [42]. However, in psoriatic patients requiring biologic therapy, with a moderate–severe disease, Kawamoto et al. reported a ILDs frequency of 2%, superior compared to ILDs prevalence of 0.01% in the referral population [8]. CT ILD pattern was not clearly reported in this study, and 50% of the patients showed psoriasis other than plaque psoriasis.

Another study reported CT patterns of 21 ILDs cases with antecedent or concomitant diagnosis of psoriasis [10]. Of the enrolled patients, 38% underwent prior or concomitant systemic treatment and 19% of them had concomitant psoriatic arthritis. UIP pattern was reported in 43% of patients, NSIP pattern in 29% and HP pattern in 9%. Moreover, in contrast with our study, organizing pneumonia pattern was reported in 19% and no patient showed PPFE pattern.

Butt et al. reported surgical pathology findings of 44 psoriatic patients with ILDs [9]. Of the patients, 61% suffered from psoriatic arthritis and 16% suffered from CTD, while only 30% of patients had not systemic immunosuppressive therapy. In this study 24 of 44 patients (55%) showed NSIP pattern at surgical pathology. However, most patients with psoriasis never require a lung biopsy, including those with evidence of ILDs. For this reason, these figures may not reflect the true spectrum of pulmonary disease in psoriatic patients, particularly in plaque psoriasis patients not under systemic treatment and without arthritis, IBD or CTD.

Considering these issues, we focused the attention in our study on patients with exclusively plaque psoriasis, finding a statistically significant higher frequency of UIP pattern in smokers than never smokers patients. Moreover, three PPFE patterns were found in plaque psoriasis patients, without significant difference between smokers and never smokers. Regarding clinical implications of our findings, it is very important for radiologists to know those peculiar CT ILD patterns that could be associated with plaque psoriasis in smokers or never smokers in order to improve the diagnostic workflow in psoriatic patients.

On the other hand, dermatologists should be aware of the possible association between ILDs and psoriasis, even in the mild-to-moderate forms, since the treatment of NSIP and HP relies on systemic corticosteroids, which cannot be used in psoriatic patients. Traditional immunosuppressants were also associated with episodes of ILDs, as well as some biological treatments, mostly TNFα inhibitors [7]. According to the physiopathological hypothesis, it may be useful to choose IL-17 inhibitors early in these patients, with the aim of controlling psoriasis and blocking possible ILD progression, including UIP, the most represented form of ILD in our sample. Furthermore, UIP seems to be related to higher mortality than NSIP in patients with ILDs and rheumatoid arthritis [43]. For this reason, dermatologists should persuade patients to stop smoking since diagnosis of psoriasis, considering that in our study UIP frequency is higher in the smokers’ group. Stopping smoking is also an important goal to control the metabolic alterations associated with psoriasis.

In our study we decided to also evaluate CT patterns in smokers, or previous smokers, and not to exclude them. Other studies decided to exclude smokers as they considered smoke a confounding factor [9,10]. In fact, smoking is an independent risk factor in the development of ILDs [9]. However, we believe that comparing a group of never smokers with exclusively plaque psoriasis and a similar group, but of smokers or previous smokers, allowed us to obtain two interesting findings. First, smokers affected by plaque psoriasis showed different CT patterns from never smokers. This can be an aid in clinical practice for a radiologist who is required to evaluate suspected ILDs in a patient with plaque psoriasis and smoker, paying more attention to the most frequent patterns for the specific patient setting. Secondly, smoking is associated with an increased risk of UIPs. This is an ulterior incentive to stop smoking, in addition to the need to reduce the cardiovascular and metabolic complications of psoriasis [23]. On the other hand, histological differences have been shown between smoking-derived and psoriasis-derived forms of ILDs. In the latter case, smoking was not the trigger but contributed to worsening respiratory symptoms [9].

Concerning mortality, data were not available for all patients, which is why we decided not to include them in this study. However, even if early diagnosis of ILDs may lead to earlier treatment, the improvement in prognosis has not been clearly defined in the literature and further studies are needed. Nowadays, early diagnosis could improve patient management, with a potential benefit for disease control [44].

Finally, to the best of our knowledge, this is the first study that evaluated ILDs in plaque psoriasis patients under topical treatment without arthritis or CTD, demonstrating a statistically significant different lung CT pattern frequency between smokers and non-smokers. Moreover, it is the first study that reported PPFE in this population. However, our study shows some limitations. First of all, the analysis was conducted on a single institution, although it is a tertiary urban academic referral hospital. Then, the analyzed cohort consisted of a relatively small sample, considering the rarity of this condition, and few patients performed lung biopsy. For these reasons, further studies are required to better understand the epidemiology of ILDs in plaque psoriasis patients.

## 5. Conclusions

In conclusion, ILDs seem to represent a new comorbidity associated with psoriasis. Dermatologists should never forget to ask their psoriatic patients about their chronic or new respiratory symptoms, in order to perform an adequate pulmonary diagnostic and therapeutic workup in collaboration with radiologist and pneumologist. Finally, dermatologists should make psoriatic patients stop smoking as soon as psoriasis is diagnosed, not only to reduce cardiovascular comorbidities, but also the risk of developing more severe forms of ILDs.

## Figures and Tables

**Table 1:** Patients’ characteristics.

Patients	Smokers n:33	Never Smokers n:32	*p*
**Age**	64.1 ± 13.9	63.7 ± 12.6	0.9032
**Sex, male**	19/33 (57.6%)	17/32 (53.1%)	0.9113
**PASI**	7.4 ± 3.5	7.3 ± 3.3	0.9236
**BSA**	5.6 ± 2.4	5.2 ± 2.5	0.4584
**sPGA**	2.7 ± 0.6	2.8 ± 0.4	0.6819
**Ps. family history**	3/33 (9.1%)	2/32 (6.3%)	0.9714
**Respiratory symptoms**	28/33 (84.8%)	24/32 (75.0%)	0.4951
shortness of breath	27/33 (81.8%)	23/32 (71.9%)	0.5113
chronic cough	23/33 (69.7%)	21/32 (65.6%)	0.9317
**FVC**	67 ± 9%	70 ± 12%	0.1744
**FEV1.0**	72 ± 8%	73 ± 11%	0.6180
**FEV1.0** **/FVC**	81 ± 3%	80 ± 3%	0.1473
**DLCO**	47 ± 11%	52 ± 15%	0.1341

PASI, psoriasis area and severity index; BSA, body surface area; sPGA, static Physician Global Assessment on a 5-point rating; Ps., psoriasis. Respiratory symptoms: chronic exertional dyspnea/shortness of breath or chronic cough. Chronic exertional dyspnea and shortness of breath were considered as a single entity. Pulmonary function tests (PFTs): FVC, forced vital capacity, FEV1.0, forced expiratory volume in 1 s, DLCO, diffusing capacity of the lungs for carbon monoxide, values expressed in % of the predicted ideal.

**Table 2:** Interstitial lung disease computed tomography patterns.

ILD CT Pattern	Smokers n:33	Never Smokers n:32	*p*
UIP	23/33 (69.7%)	13/32 (40.6%)	0.0351
NSIP	7/33 (21.2%)	12/32 (37.5%)	0.2417
HP	2/33 (6.1%)	5/32 (15.6%)	0.3990
PPFE	1/33 (3.0%)	2/32 (6.3%)	0.9782

ILD, interstitial lung disease; CT, computed tomography; UIP, usual interstitial pneumonia; NSIP, nonspecific interstitial pneumonia; HP, hypersensitivity pneumonitis; PPFE, pleuropulmonary fibroelastosis.

## Data Availability

All data are included in this manuscript.
